# Depressive disorders are associated with increased peripheral blood cell deformability: a cross-sectional case-control study (Mood-Morph)

**DOI:** 10.1038/s41398-022-01911-3

**Published:** 2022-04-08

**Authors:** Andreas Walther, Anne Mackens-Kiani, Julian Eder, Maik Herbig, Christoph Herold, Clemens Kirschbaum, Jochen Guck, Lucas Daniel Wittwer, Katja Beesdo-Baum, Martin Kräter

**Affiliations:** 1 Biopsychology, TU Dresden, Dresden, Germany; 2 Clinical Psychology and Psychotherapy, University of Zurich, Zurich, Switzerland; 3 Center for Molecular and Cellular Bioengineering, Biotechnology Center, TU Dresden, Dresden, Germany; 4 Max Planck Institute for the Science of Light & Max-Planck-Zentrum für Physik und Medizin, Erlangen, Germany; 5 Zellmechanik Dresden GmbH, Dresden, Germany; 6 Faculty of Informatics/Mathematics, HTW Dresden, Dresden, Germany; 7 Behavioral Epidemiology, TU Dresden, Dresden, Germany

**Keywords:** Depression, Physiology, Diagnostic markers

## Abstract

Pathophysiological landmarks of depressive disorders are chronic low-grade inflammation and elevated glucocorticoid output. Both can potentially interfere with cytoskeleton organization, cell membrane bending and cell function, suggesting altered cell morpho-rheological properties like cell deformability and other cell mechanical features in depressive disorders. We performed a cross-sectional case-control study using the image-based morpho-rheological characterization of unmanipulated blood samples facilitating real-time deformability cytometry (RT-DC). Sixty-nine pre-screened individuals at high risk for depressive disorders and 70 matched healthy controls were included and clinically evaluated by Composite International Diagnostic Interview leading to lifetime and 12-month diagnoses. Facilitating deep learning on blood cell images, major blood cell types were classified and morpho-rheological parameters such as cell size and cell deformability of every individual cell was quantified. We found peripheral blood cells to be more deformable in patients with depressive disorders compared to controls, while cell size was not affected. Lifetime persistent depressive disorder was associated with increased cell deformability in monocytes and neutrophils, while in 12-month persistent depressive disorder erythrocytes deformed more. Lymphocytes were more deformable in 12-month major depressive disorder, while for lifetime major depressive disorder no differences could be identified. After correction for multiple testing, only associations for lifetime persistent depressive disorder remained significant. This is the first study analyzing morpho-rheological properties of entire blood cells and highlighting depressive disorders and in particular persistent depressive disorders to be associated with increased blood cell deformability. While all major blood cells tend to be more deformable, lymphocytes, monocytes, and neutrophils are mostly affected. This indicates that immune cell mechanical changes occur in depressive disorders, which might be predictive of persistent immune response.

## Introduction

Depressive disorders including major depressive disorder (MDD) and persistent depressive disorder (PDD; formerly dysthymia) are the leading causes of disability worldwide [1]. To diagnose MDD a two-week phase is required. During this phase, at least one of the two cardinal symptoms “depressive mood” or “anhedonia” in combination with four or more of seven other symptoms (e.g., changes in appetite, insomnia/hypersomnia, increased fatigue, feelings of worthlessness) have to be present for most of the day and must cause functional impairment. PDD is diagnosed based on a period of depressive mood over 2-years in combination with at least two of six additional symptoms similar to MDD [2]. To date, physiological manifestations only play a theoretical role in diagnostics. This is due to the fact that the pathophysiology of depressive disorders remains insufficiently understood.

The two most consistent and salient physiological abnormalities are a hyperactive hypothalamus-pituitary-adrenal (HPA) axis and chronic low-grade inflammation associated with elevated cortisol and proinflammatory cytokine levels, respectively [3]. In line with this, an increased lymphocyte count has been identified in MDD and PDD [4]. Interestingly, increased levels of natural killer cells were rescued after antidepressant treatment in MDD and PDD patients suggesting a depressive disorder-dependent state of increased lymphocyte cell count. Corroborating this, in a recent study including a sample of 206 depression cases and 77 healthy controls, increased neutrophil and monocyte counts were described in cases as compared to controls [5]. These findings support a depressive disorder-dependent state of increased immune cell count pointing toward a hyperactive immune system in subjects with depressive disorders. It is suggested that the underlying mechanism leading to increased immune cell counts in depressed individuals is rooted in the effect of elevated glucocorticoid levels remodeling the actin cytoskeleton of blood cells and thereby softening leukocytes and enabling them to demarginate from the vessel wall [6].

Increased cortisol levels and chronic low-grade inflammation in individuals suffering from depressive disorders not only increase immune cell count but directly influence blood cells by crucially affecting actin cytoskeleton, lipid metabolism and composition, and cell membrane formation [6]. These processes cause softening and increased bending and destabilization of the cell [7–10], which ultimately reduces blood cell function. Therefore, we hypothesize depressive disorders to be associated with altered peripheral blood cell function, which might be represented by the cell’s morpho-rheological properties such as increased cell deformability.

Blood is a poly-disperse suspension of a number of different cell types, representing multiple functions from metabolite transport to overall blood flow. The morpho-rheological properties including cell mechanical features or cell size of each cell can be predictive of its specific physiological or pathological function [11, 12]. It was recently highlighted, that the assessment of the blood cell mechanical status, measured by cell deformability under constant shear stress, is appropriate to detect and classify human disease conditions [13]. For example, in this series of experiments, it was observed that individuals suffering from spherocytosis exhibited reduced erythrocyte deformability, which was also documented for erythrocytes exposed to *Plasmodium falciparum* the principal malaria-causing parasite [13]. By contrast, inhalation of lipopolysaccharide from *E. coli*, infecting blood in vitro with Staphylococcus aureus, having an acute lung injury, or suffering from viral respiratory tract infections or COVID-19 infection led all to increased neutrophil deformation [13, 14]. Similarly, patients with Epstein-Barr-virus infection showed increased monocyte and lymphocyte deformability [13]. This suggests that in certain disease conditions altered blood cell function may be present being measurable via blood cell mechanical properties such as cell deformability. Cell deformability has so far never been investigated in relation to depressive disorders.

Furthermore, proof-of-concept studies have used optical traps [15], atomic force microscopy [11, 12], and micropipette aspiration [16] to show immune cell mechanical alterations during physiological and pathological conditions. Most likely due to the overall predominance of erythrocytes in the blood, a correlation between blood cell mechanics and mental disorders has so far only been examined by measuring erythrocyte deformability [17, 18]. These studies have used different methods to investigate aspects of erythrocyte deformability and have identified that chronic fatigue patients have lower erythrocyte deformability than healthy controls, while children with more severe symptoms in the autism spectrum also exhibited lower erythrocyte deformability [17, 18]. However, immune cells seem to be more likely effectors of increased cortisol levels in depressed individuals through specific interactions with membrane-bound glucocorticoid receptors and also increased chronic low-grade inflammation through immune cells’ interaction with cytokines [19, 20]. Therefore, the parallel study of cell deformability of all blood cell types with respect to a particular condition provides broader insight into the underlying pathophysiology and the potential for internal replication. Thus, progress towards clinical application has yet not been achieved, potentially due to the lack of measurement throughput with only a couple of hundred cells per hour [21].

**Fig. 1:**
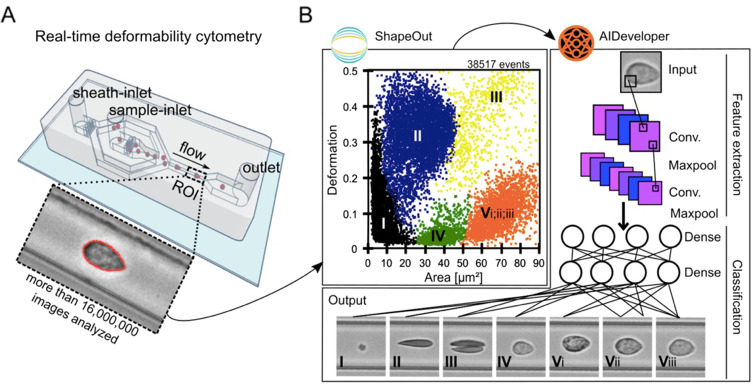
Real-time deformability cytometry and subsequent AI-based classification of blood cells. **A** A schematic illustration of an RT-DC measurement chip is shown. Whole blood was resuspended in measurement buffer (CellCarrierB), drawn in a syringe, and connected to the sample inlet. CellCarrierB was used as a sheath fluid within a second syringe and the sample and sheath were flushed through the chip at a ratio of flow rates of 1:3 under constant flow (0.06 µL/s). The chip was mounted to an inverted microscope and an image of every cell was recorded at the end of a 600 µm long constriction cannel. **B** Using ShapeOut, an open-source software tool, data were plotted. The dot-plot shows a measurement of 38,517 blood cells plotted in cell size (projected area [µm²]) and cell deformability. For better representation, the ratio of leukocytes (IV, V_i_-_iii_) to erythrocytes and thrombocytes (I–III) is artificially increased. In order to identify the different blood cell types, the images were imported to AIDeveloper an open-source software tool to train, evaluate, and apply neural networks for image classification. A neural net based on the LeNet5 architecture, readily trained for the classification of blood cells, was loaded into AID and used to classify I = thrombocytes, II = erythrocytes, III = erythrocyte doublets, IV = lymphocytes, V_i_ = eosinophils, V_ii_ = neutrophils, and V_iii_ = monocytes [24]. Finally, the mean values for cell deformability and cell size were extracted for every cell type individually.

Here, we used state-of-the-art real-time deformability cytometry (RT-DC) together with artificial intelligent-based image processing in order to overcome the throughput limitations (Fig. 1). We measured the morpho-rheological properties of more than 16 × 10^6^ single blood cells of 139 individuals at high risk for depressive disorders and matched healthy controls (HCs). RT-DC facilitates microfluidics and high-throughput imaging to assess up to 1000 cells per second. Based on the cell image, RT-DC quantifies multiple parameters including the cell’s deformability under shear stress and cell size without the need for blood preparation, like cell staining or erythrocyte depletion [22]. The observed deformability is dependent on the cell´s mechanical properties. These properties are representative of the molecular composition and the cytoskeletal state [23, 24], which are arguably the most crucial aspects to fulfill tissue and cell-specific functionality. Thus, we argue that the precise control of mechanical features of blood cells is indispensable to keep physical and psychological homeostasis. Thus, cell mechanical properties potentially comprise crucial pathophysiological information in mental disorders and particularly depressive disorders.

## Method

### Study design and setting

The study entitled mood-related morpho-rheological changes in peripheral blood cells (Mood-Morph) included a prescreening to select participants suffering from depressive disorders and healthy control subjects matched by age and sex, followed by a cross-sectional case-control study. Study visits included a clinical diagnostic interview, psychometric testing, and blood sampling. The study was approved by the local ethics committee of the Dresden University of Technology (EK182042019) and all participants gave written informed consent to participate in the study.

### Participants

**Fig. 2:**
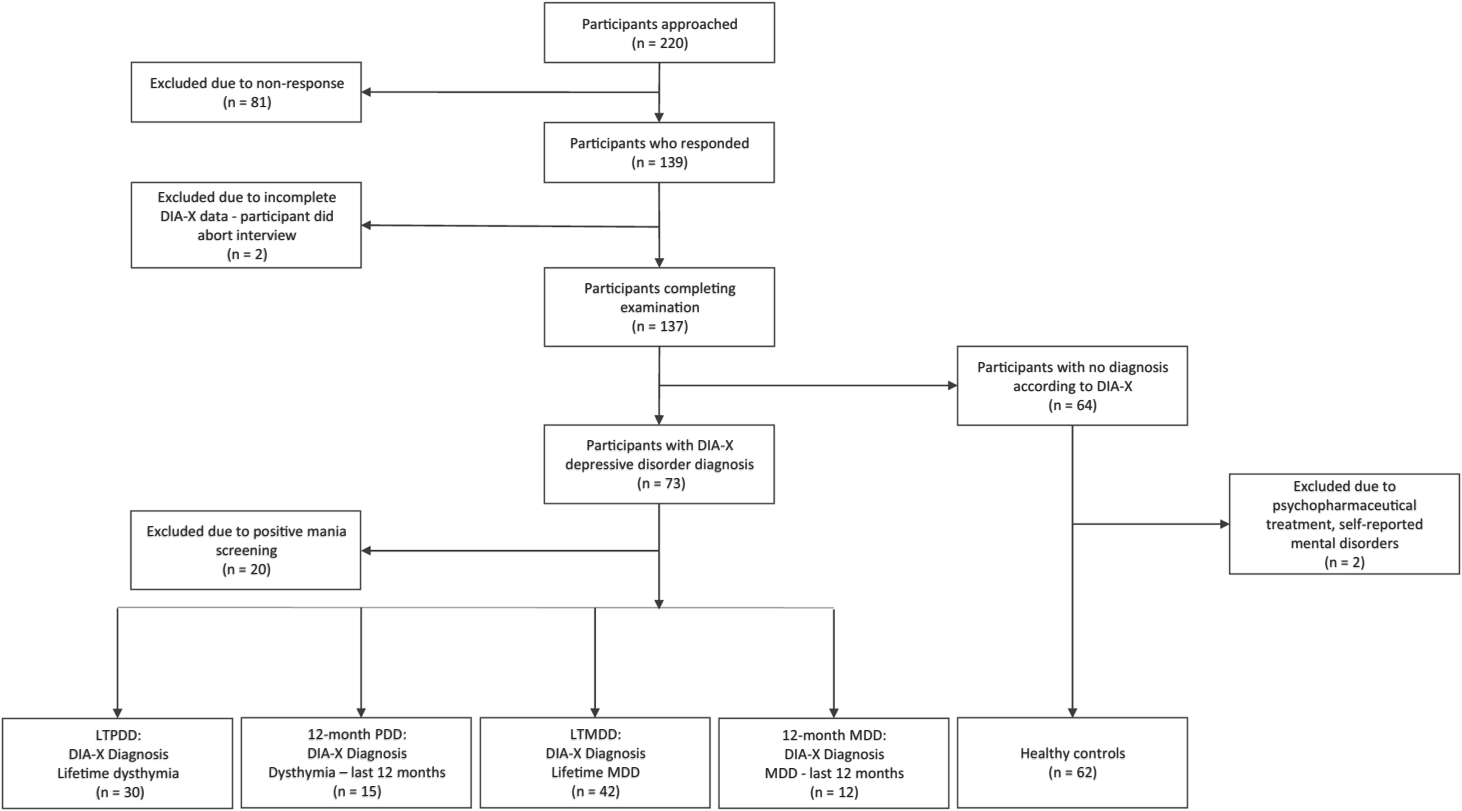
Sample flow leading to relevant case-control groups. DIA-X-5 The DIA-X-5/Composite International Diagnostic Interview (DIA-X-5/CIDI) is a standardized clinical interview for the assessment of mental disorders, LTPDD lifetime persistent depressive disorder, 12-month PDD 12-month persistent depressive disorder, LMDD lifetime major depressive disorder, 12-month MDD 12-month major depressive disorder.

Recruitment was performed from the participant pool of the large prospective cohort study on stress-related mental disorders (Dresden Burnout Study [DBS]) with respect to depression scores (measured with the Patient Health Questionnaire [PHQ-9]) in the most recent examination wave from October to December 2018. Subjects with a score >10 (high risk of depression) as well as age- and sex-matched subjects with a score <5 (low risk of depression) were invited. This procedure’s aim was to achieve a sample of participants suffering from depressive disorders and a matched sample of healthy control subjects (Fig. 2). Based on previous studies by our group that investigated the deformability of cells by real-time deformability cytometry (RT-DC) with respect to different somatic conditions [13], we used a sample size for the present study that is sufficiently powered to identify medium to large effects.

The required age for participation was between 18 and 68 years. Study participants were excluded if they suffered from a blood disease that could affect the deformability of blood cells. Potential participants contacted were informed in the study invitation and informational email that certain diseases, such as spherocytosis and, in general, diseases related to blood, precluded participation [13]. To exclude acutely infectious participants, potential participants with a cold or another infection no longer ago than two weeks were not included. In addition, participants answered a questionnaire at the beginning of the study (before the clinical diagnostic interview) to ensure that there were no physical diseases that could affect cell deformability. However, conditions that were prevalent in the population, such as hypertension, did not lead to exclusion, and the specific drug categories (e.g., for hypertension—antihypertensive drugs) were then examined in the analyses. Individuals who reported psychopharmaceutical treatment or mental disorders in the questionnaire or one was identified in the clinical interview were excluded from the healthy control group. For data analysis, individuals screening positive for any other mental disorder or for mania were excluded from the PDD and MDD groups.

### Procedures

Depressive symptom severity, as well as general health, were measured online via the DBS-homepage one week prior to examination using the depression section of the PHQ-9 and the Short Form Health Questionnaire (SF-12) [25, 26]. The PHQ is a self-report questionnaire for the assessment of common mental disorders with the PHQ-9 being the module designed for the measurement of depression severity. The SF-12 measures physical and psychological health-related quality of life.

During an examination, participants completed a subject questionnaire regarding age, sex, weight, height, medication, drug use, psychopharmacological treatment, and psychotherapeutic treatment. After completion of the questionnaire, the depression section of the Composite International Diagnostic Interview (DIA-X-5/CIDI) was conducted by trained interviewers [27]. The DIA-X-5 is a standardized clinical interview for the assessment of mental disorders. The DIA-X-5 first asks about the presence of lifetime symptoms, followed by questions about the worst lifetime episode, and finally which of these symptoms also occurred in the past 12 months. A mania-screening questionnaire, consisting of the initial questions of the DIA-X-5 mania section, was conducted to detect any lifetime (hypo-) mania symptoms. Lifetime and 12-month diagnoses were subsequently generated for PDD and MDD according to DSM-5 criteria [2]. Subsequently, a 20 µl capillary blood sample was extracted from the fingertip using a safety lancet. The blood was immediately diluted with 380 µl of measurement buffer (CellCarrierB, Zellmechanik Dresden, Germany) in a microcentrifuge tube. All subjects signed consent forms regarding data privacy, clinical data collection and saving, and blood extraction. Subjects received 15 € compensation for expenses. The duration of the examination was ~1 h. After examination, blood samples were transferred to the Department of Cellular Machines at the Biotechnology Center of the TU Dresden, where the samples were measured using an RT-DC device.

### Real-time deformability cytometry (RT-DC)

A 20 µl blood drop was taken from study participants by finger pricking using a lancet (Safety-Lancet Normal 21, Sarstedt, Nümbrecht, Germany) and harvested in a capillary (Minivette POCT, 20 µl, Sarstedt, Nümbrecht, Germany). Blood was immediately resuspended in 380 µl RT-DC measurement buffer containing 0.6% methylcellulose (CellCarrierB; Zellmechanik Dresden, Germany), maintained at room temperature, and measured within 3 h according to a protocol published elsewhere [13]. Overnight fasting of individuals is not required for this method [14, 22]. In brief, blood was flushed through a microfluidic channel constriction of 20 µm × 20 µm in cross-section (Flic20, Zellmechanik Dresden, Germany) by applying a constant flow rate. An image of every measured blood cell was taken by a high-speed camera (Fig. 1A) and besides other parameters, cell deformability and projected area (cell size) were calculated [22]. RT-DC measurements were controlled by the acquisition software Shape-In2 (Zellmechanik Dresden, Germany). The different blood cell types were classified by utilizing artificial intelligence-based image classification as published elsewhere [28] and mean values for cell deformability and cell size of every donor and blood cell type were extracted (Fig. 1B).

### Statistical analysis

Data were analyzed using R 3.4.3 [29]. Two-tailed independent *t*-tests and Mann–Whitney *U*-tests were performed to compare deformability and cell size (projected area [µm^2^]) of each blood cell type between healthy controls and depressed individuals. Data were checked for normality using Shapiro–Wilk tests and *p* values were adjusted for multiple testing using a step-down Holm–Bonferroni method to control for familywise error rate of committing type I errors [30]. Moderating confounders (sex, age, BMI, psychopharmaceutical intake, medication category) were either adjusted for or tested using two-tailed independent *t*-tests and univariate ANOVA.

## Results

### Case-control distribution for the group contrasts

A total of 139 pre-screened individuals scoring above 10 (*n* = 69) or below 5 (*n* = 70) in the PHQ-9 were examined in the study. Individuals meeting a positive screen for mania were excluded from PDD and MDD groups. Individuals reporting psychopharmaceutical treatment or mental disorders in the subject questionnaire were excluded from the healthy control group. Exclusion criteria and the DIA-X-5 diagnosis led to the following groups: Lifetime persistent depressive disorder (LTPDD) (*n* = 30), 12-month persistent depressive disorder (12-month PDD) (*n* = 15), lifetime major depressive disorder (LTMDD) (*n* = 42), 12-month major depressive disorder (12-month MDD) (*n* = 12), and healthy controls (HC) (*n* = 62). PDD and MDD groups partially overlap. Each group of participants suffering from depression was compared to an age- and sex-matched healthy control group. For detailed sample characteristics see Table 1.

**Table 1:** Sample characteristics according to different diagnostic groups.

	Lifetime PDD cases (*n* = 30)				Healthy controls (*n* = 30)				Difference*
	*n*	Minimum	Maximum	*M* (SD)	*n*	Minimum	Maximum	*M* (SD)	
**Lifetime PDD**									
Sex									1.000^a^
Male	8	..	..	..	8	..	..	..	
Female	22	..	..	..	22	..	..	..	
Age	..	22	62	49.10 (10.66)	..	22	62	49.03 (10.64)	0.962^b^
BMI	..	17.91	33.66	25.74 (3.37)	..	18.59	34.63	25.33 (4.78)	0.727^c^
Medication**	10	..	..	..	0	..	..	..	**0.002**^**a**^
PHQ-9	..	2	25	13.37 (5.6)	..	0	10	3.53 (2.47)	**0.000**^**b**^
SF-12									
Physical health	..	20.15	57.26	42.87(10.16)	..	28.04	59.77	54.07 (6.06)	**0.000**^**b**^
Mental health	..	17.57	60.24	33.82 (10.72)	..	35.51	62.12	52.48 (7.24)	**0.000**^**b**^
	**12-month PDD cases (*n*** = **15)**				**Healthy controls (*n*** = **15)**				
**12-month PDD**									
Sex									1.000^a^
Male	4	..	..	..	4	..	..	..	
Female	11	..	..	..	11	..	..	..	
Age	..	26	61	50.60 (9.75)	..	26	61	50.67 (9.56)	0.975^b^
BMI	..	19.23	31.80	26.50 (3.86)	..	18.87	33.06	25.63 (4.88)	0.594^c^
Medication**	9	..	..	..	0	..	..	..	**0.001**^**a**^
PHQ-9	..	9	25	15.60 (5.07)	..	0	7	3.13 (1.92)	**0.000**^**b**^
SF-12									
Physical health	..	20.15	57.26	38.46 (11.17)	..	28.04	59.72	52.55 (7.97)	**0.000**^**b**^
Mental health	..	21.08	45.96	29.62 (6.83)	..	35.51	62.11	52.90 (8.34)	**0.000**^**b**^

	Lifetime MDD cases (*n* = 42)				Healthy controls (*n* = 42)				Difference*
	*n*	Minimum	Maximum	*M* (SD)	*n*	Minimum	Maximum	*M* (SD)	
**Lifetime MDD**									
Sex									1.000^a^
Male	8	..	..	..	8	..	..	..	
Female	34	..	..	..	34	..	..	..	
Age	..	22	65	46.67 (11.38)	..	22	64	46.14 (11.69)	0.812^b^
BMI	..	17.57	37.24	25.35 (4.58)	..	18.59	39.10	25.39 (5.34)	0.761^b^
Medication**	14	..	..	..	0	..	..	..	**0.000**^**a**^
PHQ-9	..	1	25	10.73 (5.89)	..	0	10	3.71 (2.53)	**0.000**^**b**^
SF-12									
Physical health	..	20.15	60.47	45.45 (10.60)	..	42.13	62.77	54.89 (4.18)	**0.000**^**b,d**^
Mental health	..	21.08	60.76	40.58 (11.84)	..	24.93	62.12	52.16 (8.83)	**0.000**^**b**^
	**12-month MDD cases (*n*** = **12)**				**Healthy controls (*n*** = **12)**				
**12-month MDD**									
Sex									1.000^a^
Male	1	..	..	..	1	..	..	..	
Female	11	..	..	..	11	..	..	..	
Age	..	26	61	49.92 (12.15)	..	26	61	49.92 (12.37)	0.989^b^
BMI	..	17.63	31.80	24.43 (4.42)	..	18.87	39.10	26.47 (5.97)	0.347^c^
Medication**	7	..	..	..	0	..	..	..	**0.007**^**a**^
PHQ-9	..	9	25	14.42 (5.40)	..	1	9	3.50 (2.50)	**0.000**^**b**^
SF-12									
Physical health	..	21.63	57.26	39.13 (10.36)	..	43.55	61.83	53.98 (5.29)	**0.000**^**c,e**^
Mental health	..	21.08	50.54	31.89 (9.48)	..	24.92	62.12	52.84 (10.77)	**0.000**^**b**^

*PDD* persistent depressive disorder, *MDD* major depressive disorder, *M* mean, *SD* standard deviation.

*t-tests, χ2-tests, and Mann–Whitney *U*-tests were calculated depending on normality and scale of measurement, ** Medication: Psychopharmaceutical treatment.

^a^χ2-test.

^b^Mann–Whitney *U*-test.

^c^*t*-test.

^d^Variance heterogeneity due to Ansari exact test.

^e^Variance heterogeneity due to *F*-test.

**Fig. 3:**
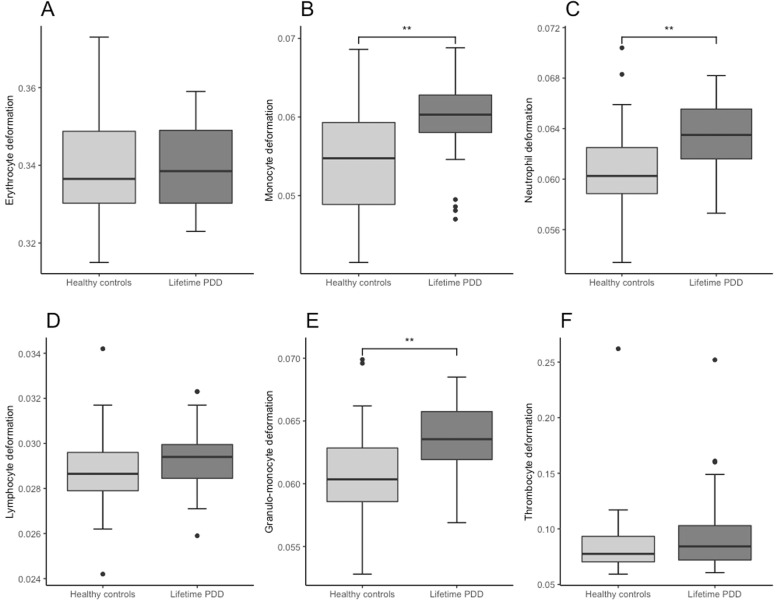
Combined boxplots for lifetime persistent depressive disorder (PDD) group and healthy controls regarding blood cell deformability. Boxplots include interquartile range (boxes), median (lines), range (whiskers), and outliers (black dots). Erythrocyte deformability (**A**), monocyte deformability (**B**), neutrophil deformability (**C**), lymphocyte deformability (**D**), granulo-monocyte deformability (**E**), thrombocyte deformability (**F**). * indicates significantly different deformability values at p < 0.05 (two-tailed), ** indicates significantly different deformability values at p < 0.01 (two-tailed).

**Fig. 4:**
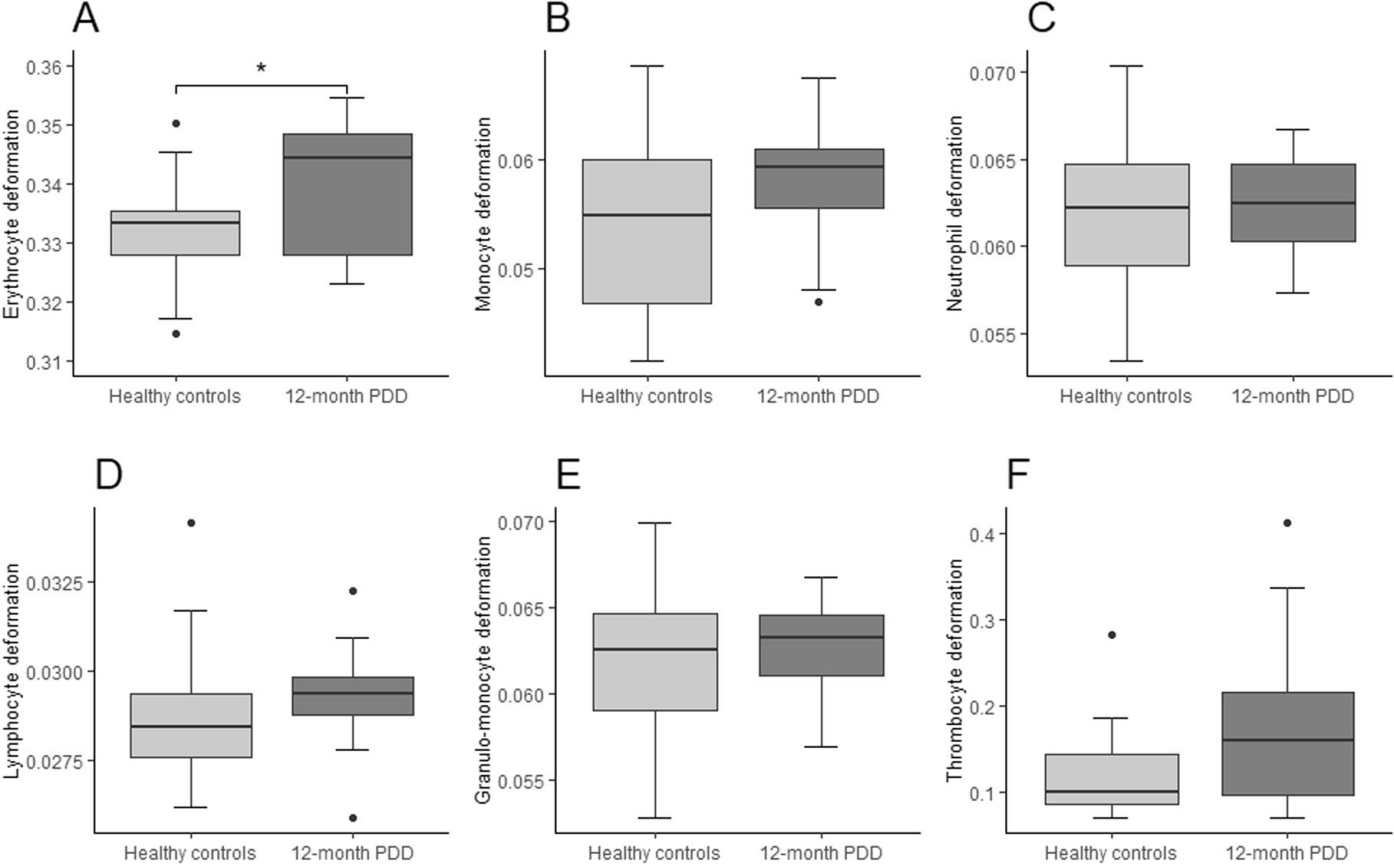
Combined boxplots for the 12-month persistent depressive disorder (PDD) group and healthy controls regarding blood cell deformability. Boxplots include interquartile range (boxes), median (lines), range (whiskers), and outliers (black dots). Erythrocyte deformability (**A**), monocyte deformability (**B**), neutrophil deformability (**C**), lymphocyte deformability (**D**), granulo-monocyte deformability (**E**), thrombocyte deformability (**F**). * indicates significantly different deformability values at p < 0.05 (two-tailed), ** indicates significantly different deformability values at p < 0.01 (two-tailed).

**Fig. 5:**
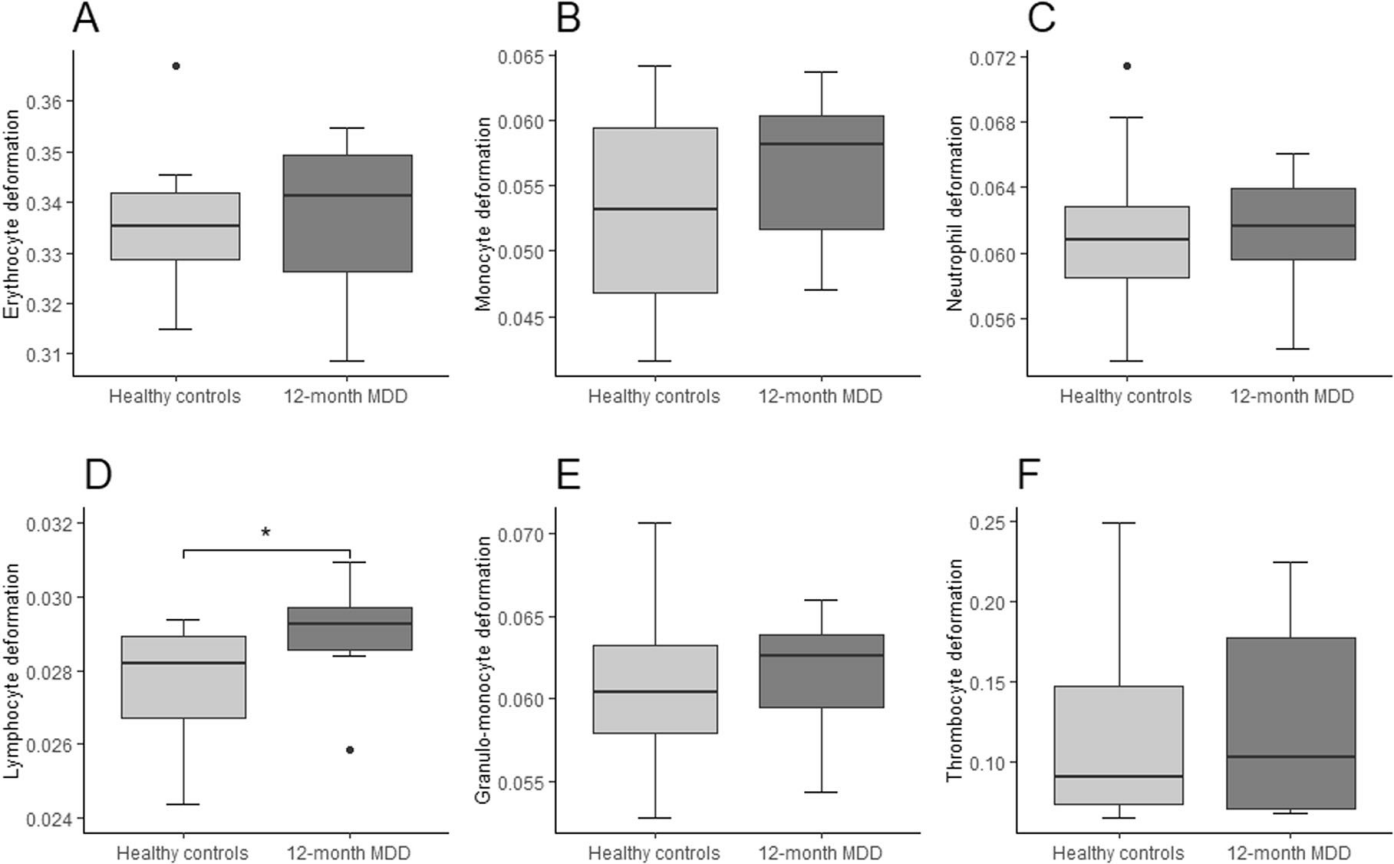
Combined boxplots for 12-month major depressive disorder (MDD) group and healthy controls regarding blood cell deformability. Boxplots include interquartile range (boxes), median (lines), range (whiskers), and outliers (black dots). Erythrocyte deformability (**A**), monocyte deformability (**B**), neutrophil deformability (**C**), lymphocyte deformability (**D**), granulo-monocyte deformability (**E**), thrombocyte deformability (**F**). * indicates significantly different deformability values at p < 0.05 (two-tailed), ** indicates significantly different deformability values at p < 0.01 (two-tailed).

**Fig. 6:**
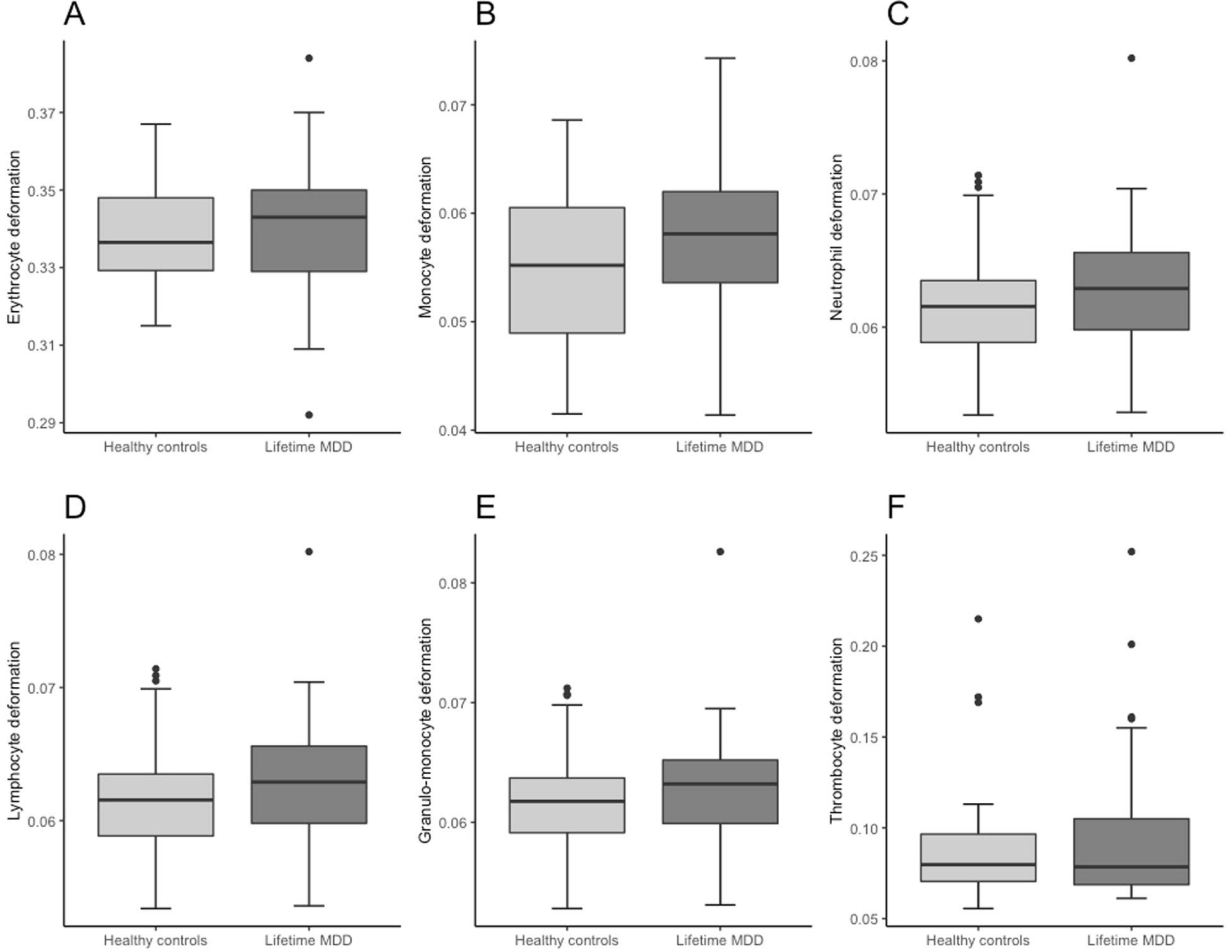
Combined boxplots for lifetime major depressive disorder (MDD) group and healthy controls regarding blood cell deformability. Boxplots include interquartile range (boxes), median (lines), range (whiskers), and outliers (black dots). Erythrocyte deformability (**A**), monocyte deformability (**B**), neutrophil deformability (**C**), lymphocyte deformability (**D**), granulo-monocyte deformability (**E**), thrombocyte deformability (**F**). * indicates significantly different deformability values at p < 0.05 (two-tailed), ** indicates significantly different deformability values at p < 0.01 (two-tailed).

While no significant difference in cell size was detected for any disease group compared to healthy controls (see Supplementary Table 1), cell deformability was altered in a disease-specific way. Two-tailed independent *t*-tests and Mann–Whitney *U*-tests showed increased cell deformability in the granulo-monocyte cell fraction, especially in monocytes (*t*(58) = 3.105, *p* = 0.003) and neutrophils (*t*(58) = 2.887, *p* = 0.005) in participants with lifetime PDD compared to healthy controls (Fig. 3). These results remained significant after applying Holm–Bonferroni corrections for multiple comparisons for both monocytes (*p* = 0.018) and neutrophils (*p* = 0.02) suggesting large effects (Cohen’s *d*: monocytes = 0.80, neutrophils = 0.74). In 12-month PDD an increased cell deformability in erythrocytes (*t*(28) = 2.082, *p* = 0.047), but no association with lymphocyte or granulo-monocyte deformability was detected (Fig. 4). 12-month MDD (Fig. 5) was associated with increased cell deformability in lymphocytes (*U*(22) = 32, *p* = 0.0224), while we found no significant effect, but only trends towards increased cell deformability in lymphocytes and myeloid cells in lifetime MDD (Fig. 6). However, significant effects vanished for the association between 12-month PDD and erythrocyte deformability (*p* = 0.282) and for the association between 12-month MDD and lymphocyte deformability (*p* = 0.132) after Holm–Bonferroni correction. For detailed statistics see Table 2.

**Table 2:** Mean cell deformability comparisons according to different diagnostic groups.

	Erythrocytes	Monocytes	Neutrophils	Lymphocytes	Granulo-Monocytes	Thrombocytes
**LTPDD (*n*** = **30)**						
*M* (SD)	0.34 (0.0102)	0.0593 (0.0053)	0.0633 (0.0029)	0.0293 (0.0015)	0.0636 (0.0029)	0.0971 (0.0411)
**HC (*n*** = **30)**						
*M* (SD)	0.338 (0.0142)	0.0545 (0.0067)	0.0608 (0.0038)	0.0287 (0.0018)	0.0609 (0.0039)	0.0865 (0.0361)
*t* value/*U* value	*t*(58) = 0.649	*t*(58) = 3.105**	*t*(58) = 2.887**	*t* (58) = 1.502	*t*(58) = 3.059**	*U* = 381.5
*p* value	0.519	**0.003**	**0.005**	0.139	**0.003**	0.311
**12-month PDD (*n*** = **15)**						
*M* (SD)	0.3394 (0.0108)	0.0579 (0.0059)	0.0624 (0.0029)	0.0293 (0.0014)	0.0626 (0.0027)	0.0964 (0.0484)
**HC (*n*** = **15)**						
*M* (SD)	0.3316 (0.0097)	0.0538 (0.008)	0.0615 (0.005)	0.0288 (0.002)	0.0617 (0.005)	0.0804 (0.0159)
*t* value/*U* value	*t*(28) = 2.082*	*t*(28) = 1.584	*t*(28) = 0.634	*t*(28) = 0.784	*t*(28) = 0.647	*U* = 94.5
*p* value	**0.047**	0.124	0.531	0.439	0.523	0.455
**LTMDD (*n*** = **42)**						
*M* (SD)	0.3396 (0.0165)	0.0573 (0.007)	0.0628 (0.0047)	0.029 (0.0018)	0.0627 (0.005)	0.0953 (0.0407)
**HC (*n*** = **42)**						
*M* (SD)	0.3376 (0.0131)	0.0554 (0.007)	0.0615 (0.0044)	0.0287 (0.0021)	0.0618 (0.0044)	0.0879 (0.0315)
*t* value/*U* value	*t*(82) = 0.619	*t*(82) = 1.26	*U* = 702	*U* = 787	*U* = 740	*U* = 829.5
*p* value	0.538	0.211	0.108	0.398	0.205	0.639
**12-month MDD (*n*** = **12)**						
*M* (SD)	0.3372 (0.0149)	0.056 (0.0057)	0.0614 (0.0035)	0.029 (0.0012)	0.0615 (0.0035)	0.0775 (0.0159)
**HC (*n*** = **12)**						
*M* (SD)	0.3355 (0.0138)	0.0531 (0.0073)	0.0611 (0.0052)	0.0277 (0.0016)	0.0611 (0.0053)	0.0796 (0.0231)
*t* value/*U* value	*t*(22) = 0.298	*t*(22) = 1.091	*t*(22) = 0.159	*U* = 32*	*t*(22) = 0.195	*U* = 70.5
*p* value	0.768	0.287	0.875	**0.022**	0.847	0.931

*LTPDD* lifetime persistent depressive disorder, *12-month PDD* 12-month persistent depressive disorder, *LTMDD* lifetime major depressive disorder, *12-month MDD* 12-month major depressive disorder.

*Indicates *p* < 0.05 (two-tailed), ** indicates *p* < 0.01 (two-tailed).

Depressive disorder and healthy control groups showed no significant differences regarding age, sex, and BMI (see Table 1). Depressive disorder and healthy control groups significantly differed in PHQ-9 scores, SF-12 scores, and usage of psychopharmaceutic treatment (see Table 1). These differences remained significant after Holm–Bonferroni correction. Psychopharmaceutical treatment was not associated with changes in cell deformability of erythrocytes (*t*(137) = −0.776, *p* = 0.439), monocytes (*t*(137) = −0.463, *p* = 0.644), neutrophils (*t*(137) = −0.603, *p* = 0.243), lymphocytes (*t*(137) = −0.096, *p* = 0.924), granulo-monocytes (*t*(137) = 1.138, *p* = 0.257), and thrombocytes (*t*(137) = −0.333, *p* = 0.3695). Subject questionnaire information on drug intake was used to create five groups according to medication: No medication (*n* = 75), psychopharmaceutical medication (*n* = 5), antihypertensive drugs (*n* = 16), thyroid dysfunction medication (*n* = 17), others (*n* = 4), and participants regularly taking in a combination of the preceding categories (*n* = 10). Univariate ANOVA showed no association between medication intake groups and cell deformability of erythrocytes (*F*_(5, 137)_ = 1.112, *p* = 0.352), monocytes (*F*_(5, 137)_ = 0.915, *p* = 0.474), neutrophils (*F*_(5, 137)_ = 0.774, *p* = 0.570), lymphocytes (*F*_(5, 137)_ = 1.051, *p* = 0.391), granulo-monocytes (*F*_(5, 137)_ = 0.904, *p* = 0.481), or thrombocytes (*F*_(5, 137)_ = 1.075, *p* = 0.377).

### Correlation analysis for diagnostic groups and matched controls

Tables 3, 4, and 5 present Pearson, Spearman, and partial correlations for the association between PHQ-9, SF-12 physical health, SF-12 mental health, and cell deformability for each blood cell type. Partial correlations correcting for age, sex, BMI, and psychopharmaceutical treatment showed for the group consisting of lifetime PDD and matched control subjects significant correlations for higher depressive symptomatology and increased cell deformability for monocytes (*r*_*p*_ = 0.306; *p* = 0.022), neutrophils (*r*_*p*_ = 0.292; *p* = 0.028), and granulo-monocytes (*r*_*p*_ = 0.293; *p* = 0.028). In addition, 12-month PDD and 12-month MDD groups with matched controls showed significant positive partial correlations for depressive symptomatology and monocytes (*r*_*p*_ = 0.389; *p* = 0.050) and erythrocytes (*r*_*p*_ = 0.452; *p* = 0.046), respectively (see Table 3). However, these significant partial correlations faded when correcting for multiple comparisons using Holm–Bonferroni correction. With respect to Pearson and Spearman correlations only Spearman correlations for neutrophils (*p* = 0.038) and granulo-monocytes (*p* = 0.025) remained significant. No significant partial correlations emerged for the SF-12 physical health scale and cell deformability of any cell type (see Table 4). For the SF-12 mental health scale, as shown in Table 5, significant partial correlations with cell deformability emerged for the group consisting of lifetime PDD subjects and controls for neutrophils (*r*_*p*_ = −0.306; *p* = 0.022) and granulo-monocytes (*r*_*p*_ = −0.304; *p* = 0.022). Negative correlations indicate higher cell deformability being associated with lower self-reported mental health. These partial correlations did not remain significant after the Holm–Bonferroni correction. No significant correlations with the SF-12 mental health scale were identified for cell deformability of the 12-month PDD and matched control group as well as the lifetime MDD and matched control group. In the 12-month MDD group and matched controls a significant partial correlation emerged for erythrocytes (*r*_*p*_ = −0.581; *p* = 0.008). This single correlation remained significant after correcting for multiple comparisons using Holm–Bonferroni correction (*p* = 0.048).

**Table 3:** Association of depressive symptoms measured by PHQ-9 and cell deformability according to different diagnostic groups.

	Erythrocytes	Monocytes	Neutrophils	Lymphocytes	Granulo-Monocytes	Thrombocytes
**LTPDD and HC (*n*** = **60)**						
Pearson	0.0228 (*p* = 0.8628)	0.3032^*^ **(*p*** **=** **0.0186)**	0.3034^*^ **(*p*** **=** **0.0184)**	0.1164 (*p* = 0.3758)	0.3166^*^ **(*p*** **=** **0.0138)**	0.117 (*p* = 0.372)
Spearman	0.0347 (*p* = 0.7924)	0.3209^*^ **(*p*** **=** **0.0124)**	0.3409^**^ **(*p*** **=** **0.0076)**	0.1481 (*p* = 0.2588)	0.3643^**^ **(*p*** **=** **0.0042)**	0.187 (*p* = 0.152)
Partial correlation	0.057 *(p* = 0.674)	0.306^*^ **(*p*** **=** **0.022)**	0.292^*^ **(*p*** **=** **0.028)**	0.071 (*p* = 0.602)	0.293^*^ **(*p*** **=** **0.028)**	0.119 (*p* = 0.381)
**12-month PDD and HC (*n*** = **30)**						
Pearson	0.3141 (*p* = 0.091)	0.3723^*^ **(*p*** **=** **0.0428)**	0.2609 (*p* = 0.1638)	0.1390 (*p* = 0.464)	0.2644 (*p* = 0.158)	0.272 (*p* = 0.146)
Spearman	0.2415 (*p* = 0.1986)	0.3618^*^ **(*p*** **=** **0.0494)**	0.2860 (*p* = 0.1256)	0.3028 (*p* = 0.1038)	0.3101 (*p* = 0.0954)	0.192 (*p* = 0.309)
Partial correlation	0.362 (*p* = 0.068)	0.389^*^ **(*p*** **=** **0.050)**	0.250 (*p* = 0.218)	0.109 (*p* = 0.596)	0.238 (*p* = 0.240)	0.319 (*p* = 0.112)
**LTMDD and HC (*n*** = **84)**						
Pearson	0.0257 (*p* = 0.8174)	0.1500 (*p* = 0.1762)	0.1261 (*p* = 0.2562)	0.0988 (*p* = 0.3744)	0.1083 (*p* = 0.3298)	0.106 (*p* = 0.339)
Spearman	0.0742 (*p* = 0.505)	0.1313 (*p* = 0.237)	0.1331 (*p* = 0.225)	0.0728 (*p* = 0.513)	0.1178 (*p* = 0.2888)	0.121 (*p* = 0.276)
Partial correlation	−0.168 (*p* = 0.136)	− 0.232^*^ **(*p*** **=** **0.038)**	−0.193 (*p* = 0.086)	−0.059 (*p* = 0.602)	−0.212 (*p* = 0.060)	0.094 (*p* = 0.409)
**12-month MDD and HC (*n*** = **24)**						
Pearson	0.2221 (*p* = 0.297)	0.345 (*p* = 0.0986)	0.2107 (*p* = 0.323)	0.2680 (*p* = 0.2054)	0.2180 (*p* = 0.3064)	0.075 (*p* = 0.729)
Spearman	0.1830 (*p* = 0.3922)	0.2950 (*p* = 0.1616)	0.2236 (*p* = 0.2936)	0.3807 (*p* = 0.0664)	0.2450 (*p* = 0.2486)	0.098 (*p* = 0.649)
Partial correlation	0.452^*^ **(*p*** **=** **0.046)**	0.263 (*p* = 0.262)	0.320 (*p* = 0.168)	0.190 (*p* = 0.422)	0.290 (*p* = 0.214)	0.164 (*p* = 0.488)

Partial correlations corrected for age, sex, BMI, and psychopharmaceutical treatment.

*LTPDD* lifetime persistent depressive disorder, *12-month PDD* 12-month persistent depressive disorder, *LTMDD* lifetime major depressive disorder, *12-month MDD* 12-month major depressive disorder.

^*^Indicates *p* < 0.05 (two-tailed).

^**^Indicates *p* < 0.01 (two-tailed).

**Table 4:** Association of physical health measured by SF-12 and cell deformability according to different diagnostic groups.

	Erythrocytes	Monocytes	Neutrophils	Lymphocytes	Granulo-Monocytes	Thrombocytes
**LTPDD and HC (*n*** = **60)**						
Pearson	0.0112 (*p* = 0.396)	−0.238 (*p* = 0.066)	−0.173 (*p* = 0.186)	−0.167 (*p* = 0.202)	−0.182 (*p* = 0.164)	−0.059 (*p* = 0.654)
Spearman	0.169 (*p* = 0.096)	−0.268* **(*p*** **=** **0.038)**	−0.216 (*p* = 0.098)	−0.202 (*p* = 0.0122)	−0.264* **(*p*** **=** **0.042)**	−0.162 (*p* = 0.217)
Partial correlation	0.063 *p* = 0.642)	−0.185 (*p* = 0.172)	−0.220 (*p* = 0.104)	−0.033 (*p* = 0.812)	−0.210 (*p* = 0.120)	−0.068 (*p* = 0.619)
**12-month PDD and HC (*n*** = **30)**						
Pearson	−0.173 (*p* = 0.360)	−0.355 (*p* = 0.054)	−0.115 (*p* = 0.546)	−0.157 (*p* = 0.406)	−0.127 (*p* = 0.504)	−0.266 (*p* = 0.155)
Spearman	−0.095 (*p* = 0.616)	−0.367* **(*p*** **=** **0.046)**	−0.073 (*p* = 0.702)	−0.249 (*p* = 0.184)	−0.102 (*p* = 0.590)	−0.385* **(*p*** **=** **0.036)**
Partial correlation	−0.196 (*p* = 0.169)	−0.294 (*p* = 0.144)	−0.157 (*p* = 0.444)	−0.074 (*p* = 0.720)	−0.151 (*p* = 0.462)	−0.339 (*p* = 0.090)
**LTMDD and HC (*n*** = **84)**						
Pearson	0.183 (*p* = 0.096)	−0.146 (*p* = 0.184)	−0.014 (*p* = 0.902)	−0.247* **(*p*** **=** **0.024)**	−0.013 (*p* = 0.904)	−0.006 (*p* = 0.957)
Spearman	0.140 (*p* = 0.204)	−0.90 (*p* = 0.418)	−0.053 (*p* = 0.636)	−0.218* **(*p*** **=** **0.046)**	−0.079 (*p* = 0.478)	−0.005 (*p* = 0.961)
Partial correlation	0.134 (*p* = 0.236)	− 0.111 (*p* = 0.328)	− 0.007 (*p* = 0.950)	−0.172 (*p* = 0.126)	0.004 (*p* = 0.972)	−0.002 (*p* = 0.985)
**12-month MDD and HC (*n*** = **24)**						
Pearson	0.155 (*p* = 0.468)	0.345 (*p* = 0.099)	−0.153 (*p* = 0.476)	−0.458* **(*p*** **=** **0.018)**	−0.196 (*p* = 0.358)	−0.089 (*p* = 0.679)
Spearman	0.140 (*p* = 0.516)	0.2950 (*p* = 0.162)	−0.158 (*p* = 0.460)	−0.473* **(*p*** **=** **0.020)**	−0.260 (*p* = 0.220)	−0.041 (*p* = 0.848)
Partial correlation	−0.079 (*p* = 0.720)	−0.331 (*p* = 0.154)	−0.214 (*p* = 0.366)	−0.305 (*p* = 0.192)	−0.213 (*p* = 0.366)	−0.058 (*p* = 0.809)

Partial correlations corrected for age, sex, BMI, and psychopharmaceutical treatment.

*LTPDD* lifetime persistent depressive disorder, *12-month PDD* 12-month persistent depressive disorder, *LTMDD* lifetime major depressive disorder, *12-month MDD* 12-month major depressive disorder.

*Indicates *p* < 0.05 (two-tailed), ** indicates *p* < 0.01 (two-tailed).

**Table 5:** Association of mental health measured by SF-12 and cell deformability according to different diagnostic groups.

	Erythrocytes	Monocytes	Neutrophils	Lymphocytes	Granulo-Monocytes	Thrombocytes
**LTPDD and HC (*n*** = **60)**						
Pearson	−0.060 (*p* = 0.648)	−0.184 (*p* = 0.160)	−0.335** **(*p*** **=** **0.008)**	−0.132 (*p* = 0.314)	−0.342** **(*p*** **=** **0.008)**	−0.160 (*p* = 0.223)
Spearman	−0.071 (*p* = 0.588)	−0.162 (*p* = 0.216)	−0.342** **(*p*** **=** **0.008)**	−0.139 (*p* = 0.288)	−0.358** **(*p*** **=** **0.002)**	−0.241 (*p* = 0.063)
Partial correlation	−0.080 *(p* = 0.560)	−0.192 (*p* = 0.156)	−0.306* **(*p*** **=** **0.022)**	−0.135 (*p* = 0.320)	−0.304* **(*p*** **=** **0.022)**	−0.164 (*p* = 0.226)
**12-month PDD and HC (*n*** = **30)**						
Pearson	−0.292 (*p* = 0.116)	−0.283 (*p* = 0.130)	−0.355 (*p* = 0.054)	−0.212 (*p* = 0.262)	−0.335 (*p* = 0.054)	−0.276 (*p* = 0.140)
Spearman	−0.227 (*p* = 0.228)	−0.221 (*p* = 0.240)	−0.337 (*p* = 0.068)	−0.320 (*p* = 0.084)	−0.336 (*p* = 0.070)	−0.196 (*p* = 0.301)
Partial correlation	−0.314 (*p* = 0.118)	−0.295 (*p* = 0.144)	−0.330 (*p* = 0.100)	−0.225 (*p* = 0.268)	−0.321 (*p* = 0.110)	−0.284 (*p* = 0.160)
**LTMDD and HC (*n*** = **84)**						
Pearson	−0.111 (*p* = 0.314)	−0.121 (*p* = 0.274)	−0.146 (*p* = 0.186)	−0.081 (*p* = 0.466)	−0.127 (*p* = 0.252)	−0.169 (*p* = 0.127)
Spearman	−0.141 (*p* = 0.202)	−0.076 (*p* = 0.492)	−0.180 (*p* = 0.102)	−0.056 (*p* = 0.616)	−0.135 (*p* = 0.222)	−0.195 (*p* = 0.077)
Partial correlation	−0.066 (*p* = 0.558)	−0.131 (*p* = 0.248)	−0.118 (*p* = 0.298)	−0.090 (*p* = 0.426)	−0.107 (*p* = 0.344)	−0.153 (*p* = 0.179)
**12-month MDD and HC (*n*** = **24)**						
Pearson	−0.427* **(*p*** **=** **0.038)**	−0.191 (*p* = 0.370)	−0.255 (*p* = 0.230)	−0.150 (*p* = 0.486)	−0.250 (*p* = 0.238)	−0.241 (*p* = 0.256)
Spearman	−0.381 (*p* = 0.066)	−0.104 (*p* = 0.628)	−0.263 (*p* = 0.214)	−0.338 (*p* = 0.106)	−0.235 (*p* = 0.270)	−0.221 (*p* = 0.299)
Partial correlation	−0.581** **(*p*** **=** **0.008)**	−0.127 (*p* = 0.592)	−0.334 (*p* = 0.150)	−0.185 (*p* = 0.434)	−0.309 (*p* = 0.186)	−0.405 (*p* = 0.077)

Partial correlations corrected for age, sex, BMI, and psychopharmaceutical treatment.

*LTPDD* lifetime persistent depressive disorder, *12-month PDD* 12-month persistent depressive disorder, *LTMDD* lifetime major depressive disorder, *12-month MDD* 12-month major depressive disorder.

*Indicates *p* < 0.05 (two-tailed), **indicates *p* < 0.01 (two-tailed).

## Discussion

To our knowledge, this is the first study providing insights into the association between depressive disorders and cell morpho-rheological features of all major blood cell types. Our results suggest depressive disorders and in particular PDD to be associated with an overall increase in blood cell deformability, while for cell size no difference was observed. Hereby, the most consistent differences were found in lymphocytes, monocytes, and neutrophils highlighting the impact of depressive disorders on the mechanical properties of primary immune cells. However, correction for multiple testing highlights differences in cell deformability in the granulo-monocyte cell fraction and neutrophils in individuals with lifetime PDD compared to healthy controls to be most pronounced.

Morpho-rheological assessment of blood cells can provide crucial health status information, as changes in the cell’s mechanical constitution are associated with physiological or pathological function [13]. Thus, increased blood cell deformability in individuals with depressive disorders compared to controls (see Figs. 3–6 and Table 3) provides novel insight into the pathophysiology of depressive disorders. However, due to the complex interplay between morpho-rheological features and cell function, we can only speculate on the underlying causal link. A large body of evidence indicates depressive disorders to be associated with increased HPA-activity resulting in elevated cortisol levels [31]. Moreover, chronic low-grade inflammation with increased levels of proinflammatory cytokines and proteins such as interleukin-6 and C-reactive protein were described [32]. Increased immune cell deformability in depressed patients might be a direct response to elevated cortisol levels, which induce actin cytoskeleton reorganization and thereby increase cell deformability [6]. Additionally, an acute immune activation with lipopolysaccharide together with an increase in proinflammatory markers were shown to increase monocyte deformability [16], suggesting the often described chronic low-grade inflammation in depressed individuals contributes to increased leukocyte deformability. Furthermore, elevated glucocorticoid levels and inflammatory markers are suggested to influence the general lipid composition leading to impaired membrane formation, stability, and increased membrane bending and destabilization [7]. Supporting this line of research, individuals exhibiting increased inflammatory signaling such as healthy individuals after inhalation of lipopolysaccharide from *E. coli*, or individuals suffering from an acute lung injury, viral respiratory tract infections, or Epstein-Barr-virus infection show all increased neutrophil, monocyte, or lymphocyte deformation measured with RT-DC [13]. Furthermore, in the acute phase of a COVID-19 infection, with elevated levels of cytokines or even cytokine storm, neutrophils showed higher deformability during the acute phase but also 7 months after acute symptoms indicating that an activated cell state can also be identified in the long term [14].

Hyperactivity of the HPA-axis, chronic low-grade inflammation, and disturbed lipid composition combined resulting in increased blood cell deformability potentially lead to overall reduced integrity and altered functionality of blood cells [6, 7, 16, 23]. Thus, the association of increased cell deformability observed in depressive disorders is in accordance with current pathophysiological models of depressive disorders. In addition, Lynall et al. (2019) reported elevated numbers of immune cells in depressed individuals compared to controls. It is known that increased levels of glucocorticoids and catecholamines result in increased white blood cell count, as cells demarginate from the vessel walls. Interestingly, these observations were recently associated with cellular softening [6]. In our study, elevated levels of circulating white blood cells in individuals suffering from depressive disorders cannot be confirmed, presumably due to the smaller sample size and the resulting lower power to detect the relatively small differences in immune cell count.

On the other hand, we also found increased erythrocyte deformability in individuals with current PDD. Tight control of homeostatic erythrocyte deformability is arguable of high importance in order to provide passage through narrow capillaries and tissue oxygen supply, critical for various organs including the central nervous system. Whether an altered lipid composition interferes with erythrocyte oxygen transport due to increased erythrocyte deformability or whether increased erythrocyte deformability is a body response to keep oxygen supply constant needs to be further examined. However, since the relation between erythrocyte deformability and current PDD does not survive correction for multiple testing, these findings need further replication and should be interpreted with caution. Nevertheless, our results highlight altered blood cell morpho-rheological properties in depressed patients to play a critical role in the pathophysiological processes; however, to what extent blood cell deformability is involved in the disease progression is yet far from understood. Furthermore, we did not identify differences in cell size of any of the investigated blood cells underlining the potential to readout cell deformability as an indicator for altered cell function. Importantly, since participants were not drug naïve, the potential association of specific medication types such as psychopharmacological agents, thyroid dysfunction medication, or antihypertensives with cell deformability was examined. No association was identified for cell deformability with any specific medication type suggesting that depressive disorders and not the examined medication types affect cell deformability.

When the correlation analysis is considered, it is noticeable that a consistent pattern emerges for the correlations between cell deformability and the PHQ-9 and the SF-12 mental health scale, while no correlations appear for cell deformability and the SF-12 physical health scale. However, it is important to emphasize that the significant correlations found do not withstand correction for multiple testing and should therefore be interpreted with caution. Nevertheless, comparable to the group comparisons, it appears that the strongest positive correlations between cell deformability and depressive symptomatology emerge in the group of lifetime PDD and HCs. Although, in general, in all groups and for all cell types positive correlations emerge, only for the lifetime PPD-HCs-subsample significant correlations were detected. More specifically, only for monocytes, neutrophils, and granulo-monocytes significant correlations between cell deformability and depressive symptoms or impaired mental health were observed suggesting these immune cells to be the most sensitive cells to react to depressive disorders with morpho-rheological changes.

Another point to be considered is that there are different ways to assess cell deformability. While the applied RT-DC method represents a versatile tool to measure specifically blood cell deformability on short time scales [13, 22], other approaches provide important insights into cell mechanical properties on longer time scales. A recent study reports reduced erythrocyte deformability in 16 patients with myalgic encephalomyelitis/chronic fatigue syndrome compared to age-matched healthy controls [17]. They examined how long erythrocytes take to cross a 5 μm × 5 μm channel at a negative pressure of −13.79 kPa. The cells deform tactile to fit through the channel, which took ~13 ms. This is more than three times as long as cells are deformed in RT-DC. Thus, in the study by Saha et al. (2019), the cell’s viscous properties might play a more pronounced role in order to pass through the channel compared to RT-DC. Interestingly, the erythrocytes derived from myalgic encephalomyelitis / chronic fatigue syndrome patients were found to be larger compared to healthy controls. Indicating again a disease-specific alteration of the morpho-rheological features. A similar report applying centrifugation of erythrocytes through a filter of 5 µm pores quantifies erythrocyte deformability as the ratio of filtered erythrocytes to initial cell number. They examined 54 children with autism spectrum disorders and identified impaired erythrocyte deformability associated with more severe restricted and repetitive symptomatology [18].

### Strengths and limitations

Strengths of our work are the pioneering character of the blood cell deformability measurement carried out using RT-DC in individuals with depressive disorders and age- and sex-matched healthy controls and the acquisition of an average of 115,000 cell images of each of the 139 subjects representing a yet not achieved large dataset. For fast, automated cell classification of the resulting dataset of over 16 million images, an artificial intelligence-based analysis was leveraged (Fig. 1). It needs to be noted, that blood samples were immediately measured using RT-DC within a time frame of 3 h after sampling since longer storage times or freezing of the cells could potentially alter cell deformability properties. In addition, the standardized clinical evaluation of the individuals provides the highest level of diagnostic validity and reliability. A limitation of our study is the fact that the participants were not drug naïve. However, since we intended to investigate depressive disorders in general including individuals with a lifetime or current PDD or MDD diagnosis, it is very difficult to include drug naïve individuals only. Therefore, in our analyses, we consistently controlled for the potential influence of medication on cell deformability. Another limitation is that PDD and MDD groups partially overlap raising the question of whether findings are related to the combination of disorders, the general severity of the symptoms of depression, or to chronicity. Further, the relatively low number of male individuals in our study renders a generalization to the male population difficult.

## Conclusions

To conclude, this study provides to our knowledge the first evidence of a relationship between peripheral blood cell deformability and depressive disorders. As the pathophysiology of depressive disorders is only poorly understood, and HPA-hyperactivity and chronic low-grade inflammation represent landmarks of the current pathophysiological model, our results further point toward a persistently activated immunity in depressive disorders. In combination with altered lipid metabolism and blood cell membrane assembly, cell functional changes mediated by cytoskeletal adaptations are very likely to occur. In agreement with other reports, we found, that these cell functional changes can be detected disease-specific by morpho-rheological measurements, potentially leading to a co-diagnostic marker. Thus, our study broadens the understanding of the current physiological underlying causes of depressive disorders in blood cells and delivers a clinical-grade method to assess erythrocyte and immune cell functionality. Future research will be needed to confirm our findings in larger cohorts, in order to render the discriminant potential of cell morpho-rheological properties in depressive disorders more specific. Moreover, the potential to reverse increased peripheral blood cell deformability might be harnessed to develop new pharmacological treatments restoring optimal levels of cell deformability, and cell function and thereby reducing depressive burden.

## Supplementary information

Supplemental Material

## Data Availability

The anonymized data will be made available to all interested parties upon request.

## Code availability

The anonymized code will be made available to all interested parties upon request.
